# *Citrus maxima* and tea regulate AMPK signaling pathway to retard the progress of nonalcoholic fatty liver disease

**DOI:** 10.29219/fnr.v66.7652

**Published:** 2022-06-10

**Authors:** Shuai Wen, Ran An, Zhi-Gang Li, Zhao-Xiang Lai, Dong-Li Li, Jun-Xi Cao, Ruo-Hong Chen, Wen-Ji Zhang, Qiu-Hua Li, Xing-Fei Lai, Shi-Li Sun, Ling-Li Sun

**Affiliations:** 1 Tea Research Institute, Guangdong Academy of Agricultural Sciences / Guangdong Key Laboratory of Tea Resources Innovation & Utilization, Guangzhou, China; 2 School of Biotechnology and Health Sciences, Wuyi University, Jiangmen, China; 3 International Healthcare Innovation Institute (Jiangmen), Jiangmen, China

**Keywords:** nonalcoholic fatty liver disease, tea, Citrus maxima, AMPK

## Abstract

**Background:**

Nonalcoholic fatty liver disease (NAFLD) is a chronic metabolic disease that easily induces hepatitis, cirrhosis, and even liver cancer. The long-term use of NAFLD therapeutic drugs produces toxicity and drug resistance. Therefore, it is necessary to develop high efficiency and low-toxicity active ingredients to alleviate NAFLD.

**Objective:**

This study aimed to reveal the role and mechanism of a new functional food CMT in alleviating NAFLD.

**Results:**

In the ob/ob fatty liver mice models, the CMT extracts significantly inhibited the weight gain of the mice and reduced the accumulation of white fat. The anatomical and pathological results showed that CMT relieved fatty liver in mice and reduced excessive lipid deposition and inflammatory infiltration. Serological and liver biochemical indicators suggest that CMT reduced dyslipidemia and liver damage caused by fatty liver. CMT obviously activated the adenosine 5′-monophosphate-activated protein kinase (AMPK)/acetyl-coA carboxylase (ACC) and AMPK/fatty acid synthase (FAS) signaling pathways, promoted fat oxidation, and inhibited synthesis. Moreover, CMT regulated the expression of inflammatory factors to relieve hepatitis caused by NAFLD.

**Conclusion:**

The study explained the role and mechanism of CMT in alleviating NAFLD and suggested that the active ingredients of CMT might be beneficial in NAFLD therapy.

## Popular scientific summary

- Ob/ob mouse, a natural nonalcoholic fatty liver disease (NAFLD) model, after 9 weeks of gavage treatment with *Citrus maxima* and tea (CMT), their body weight, Lee’s index, white fat, liver index, and lipid deposition were significantly reduced.
- Serological and liver biochemical indicators have shown that TC, TG, LDL-C, FFA and HDL-C in obese mice were obviously regulated by CMT.
- CMT significantly promoted fat oxidation via the AMPK/ACC signaling pathway and inhibited fat synthesis via AMPK/FAS signaling pathways.

Nonalcoholic fatty liver disease (NAFLD) is a metabolic disease that is closely related to obesity and diabetes (1). A majority of the patients reside in developed countries, and the disease is related to the long-term consumption of diets containing excessive sugar, fat, and high-calorie foods (2). Although NAFLD is a chronic disease, simple fatty liver in the initial stage has no obvious effects on life and health. However, in the middle and late stages, it may change to complicated liver diseases, such as fatty hepatitis, liver fibrosis, liver failure, cirrhosis, and even liver cancer (3, 4).

The current treatments for NAFLD include basic treatment (such as adjusting dietary structure and strengthening exercise), drug treatment (such as alleviating insulin resistance and regulating lipid metabolism), and surgical treatment (such as reducing stomach volume and liver transplantation) (5–7). Many chemical drugs, such as metformin and statins, used to enhance insulin sensitivity and promote lipid metabolism have been used clinically (8, 9). However, these chemical drugs have potential toxic and side effects, which cause serious toxicity upon the patients’ long-term use, and may even aggravate the disease (10). In addition, the long-term use of these drugs will cause drug resistance and deteriorate the drugs’ properties. Therefore, searching for high-efficiency and low-toxicity active ingredients to relieve NAFLD from natural products and dietary foods has attracted increasing attention recently. For example, the natural ingredient oxymatrine regulates lipid metabolism by regulating the NAD-dependent protein deacetylase sirtuin-1 (SIRT1)/adenosine 5′-monophosphate-activated protein kinase (AMPK) signaling pathway and its downstream proteins, thereby alleviating NAFLD (11). Tea and its active ingredients, tea polyphenols, and (−)-epigallocatechin-3-gallate (EGCG) have the potential to prevent and treat NAFLD (12). Hesperidin has lipid-lowering and anti-inflammatory effects in the treatment of NAFLD (13).

*Citrus maxima* cv. T. is used in China as an effective product for daily consumption, such as for relieving cough, reducing phlegm, and promoting gastrointestinal function (14). Tea is one of the three major beverages in the world and has a long history of planting and drinking. According to the processing technology, tea can be divided into nonfermented tea, semi-fermented tea, and fully fermented tea (15). Previous studies have confirmed that tea has many advantages such as anticancer properties and weight loss effects (16–18). In this study, nonfermented tea (green tea), semi-fermented tea (yellow tea), or fully fermented tea (black tea) was put into the peel of *Citrus maxima* cv. T. to make a new functional drink (called *Citrus maxima* and tea [CMT]) as the experimental material. Using ob/ob mice as the research model, we studied the relieving effects and molecular mechanisms of three types of CMT on NAFLD.

## Materials and methods

### Preparation of CMT

*Citrus maxima* and black tea (CB), *Citrus maxima* and yellow tea (CY), and *Citrus maxima* and green tea (CG) were all provided by Tea and health laboratory in Tea Institute of Guangdong Academy of Agricultural Sciences. CB, CY, and CG were, respectively, combined from *Citrus maxima* cv. T. and Yinghong No. 9 black tea, yellow tea, and green tea. Specifically, the tea after kneading was filled into the *Citrus maxima* cv. T., which the pulp was dug out, and then followed by fermented drying, sallow drying, or direct drying. The ratio of peel to tea was about 1:1.5.

### Preparation and monomer detection of tea extract

The grated tea was mixed with 20 times the volume of boiling water and boiled for 30 min, repeated three times. The extract was filtered, concentrated, and freeze dried to obtain CMT freeze-dried powder. The biochemical composition and monomer composition analysis of CMT have been measured and reported before (19).

### Grouping and administration of mice

Male C57BL/6J mice and male ob/ob mice were purchased in Beijing Huafukang Biotechnology Co., Ltd. and kept in a specific pathogen-free (SPF) animal room. The control (CT) group consisted of eight C57BL/6J male mice. Thirty-two male ob/ob mice were randomly divided into ob/ob model (MD) group, CB, CY, and CG group, with eight in each group. Each administration group was given 600 mg/kg·BW corresponding tea extract every day. The control group and the model control group were given an equal volume of pure water every day. The mice were given intragastric administration twice a day for 9 weeks. Weight, diet consumption, and water intake were continuously recorded. Lee’s index was calculated as follows: *Lee’s index* = *weight* (g) ^ (1/3) × 10/*body length* (mm).

### Mice tissues collection

Mice whole blood were allowed to stand at room temperature for 2–4 h, centrifuged at 1,000 g/min for 20 min, and the serums were carefully collected. The liver was photographed and weighed and fixed in 4% paraformaldehyde. Epididymal fat, intestinal fat, perirenal fat, and subcutaneous fat were taken out and weighed. Tissues and serums were frozen at −80°C.

### Biochemical indicator detection

High-density lipoprotein cholesterol (HDL-C), total cholesterol (TC), low-density lipoprotein cholesterol (LDL-C), triglyceride (TG), alanine aminotransferase (ALT), free fatty acid (FFA), and asparagus transaminase (AST) detection kits were purchased from Nanjing Jiancheng Institute of Bioengineering Co., Ltd. 10% tissue homogenate was prepared from liver tissue with normal saline, and the above-mentioned biochemical indicators of liver tissues homogenate and serum were measured according to the manufacturer’s instructions.

### Hematoxylin-eosin staining

The fixed liver tissues were cut into tissue blocks with a thickness of 2–3 mm and embedded in paraffin. The tissue block was cut into 4-μm thick sections with a paraffin microtome (Leica, Switzerland). The sections were deparaffinized twice with xylene and rehydrated with 100, 95, 80, and 70% ethanol gradients. Then the sections were stained with hematoxylin (Shanghai Beyotime Biotechnology) for 10 min and rinsed with tap water for 10 min. The sections were stained with eosin (Shanghai Beyotime Biotechnology) for 40 sec, dehydrated with 95 and 100% ethanol, made transparent twice with xylene, sealed with neutral resin, and finally observed and photographed with a microscope (Olympus, Japan).

### Immunohistochemistry

The sections were deparaffinized and rehydrated according to the method described as hematoxylin-eosin (HE) staining. The sections were inactivated by 3% H_2_O_2_ (Solarbio), and then ethylene diamine tetraacetic acid (EDTA) (Solarbio) was used to repair the antigen. The sections were blocked with 5% goat serum (Solarbio) for 30 min and then incubated with the corresponding primary antibody at 4°C overnight. The primary antibodies used include p-AMPKα (CST, 2535), p-acetyl-coA carboxylase (ACC) (CST, 11818), carnitine palmitoyltransferase 1 (CPT-1) (Abcam, ab128568), fatty acid synthase (FAS) (CST, 3180), and CD36 (protientech, 66395). Then the sections were incubated with the secondary antibody for 1 h and with the streptavidin–biotin complex (SABC) reagent (Shanghai Beyotime Biotechnology) reacted for 30 min. Then the sections were developed with diaminobenzidine (DAB) (Shanghai Beyotime Biotechnology) for 2–5 min and hematoxylin (Shanghai Beyotime Biotechnology) counterstained for 90 sec. Finally, the sections were dehydrated by ethanol gradient, treated with xylene, mounted with neutral resin, and observed under a microscope and photographed.

### Western blot analysis

The liver tissues were homogenized in RIPA (radio immunoprecipitation assay) lysate (Shanghai Beyotime Biotechnology), left standing in an ice bath for 1 h, and centrifuged at 4°C for 20 min at the highest speed. The supernatant after centrifugation was used as a protein sample, and the protein concentration was calculated using the bull serum albumin (BSA) kit (Shanghai Beyotime Biotechnology). Then 20 μL of each protein sample was separated by electrophoresis, including 80 V concentrated gel electrophoresis and 110 V separation gel electrophoresis. The gels were transferred to the polyvinylidene fluoride (PVDF) membranes (Millipore) at a constant current of 275 mA for 70 min. After the membranes were blocked with 5% skimmed milk (Bio-FROXX) for 2 h, they were incubated with the corresponding primary antibody at 4°C overnight. Primary antibodies include p-AMPKα (CST, 2535), AMPKα (CST, 2532), inhibiting kappa B kinase α (IKKα) (CST, 2682), p-ACC (CST, 11818), ACC (CST, 3676), interleukin-6 (IL-6) (Santa Cruz, SC-1265), FAS (CST, 3180), Actin (CST, 4970), cyclooxygenase-2 (COX-2) (Boster, BA0738), CPT-1 (Abcam, ab128568), inducible nitric oxide synthase (iNOS) (Abcam, ab15323), TNF-α (Abcam, ab6671), and CD36 (protientech, 66395). Then the membranes were incubated with the secondary antibody (KPL, 074-1506 or 074-1806) for 50 min, the developer (Tanon 5200) was reacted in the dark for 2 min, and the chemiluminescence gel imaging system (Tanon 5200) was used for imaging. Image J software was used to quantitatively analyze the gray level of bands.

### Statistical analysis

All data were expressed as mean ± standard deviation (Mean ± SD), and each experiment was repeated at least four times under the same conditions. GraphPad Prism 7.0 software was used for data analysis. One-way analysis of variance (ANOVA) was performed to analyze the data between the tea samples. Different lowercase letters indicated significant differences at the *P* < 0.05.

## Results

### CMT affects weight, diet consumption, water intake, and Lee’s index of mice

**Fig. 1:**
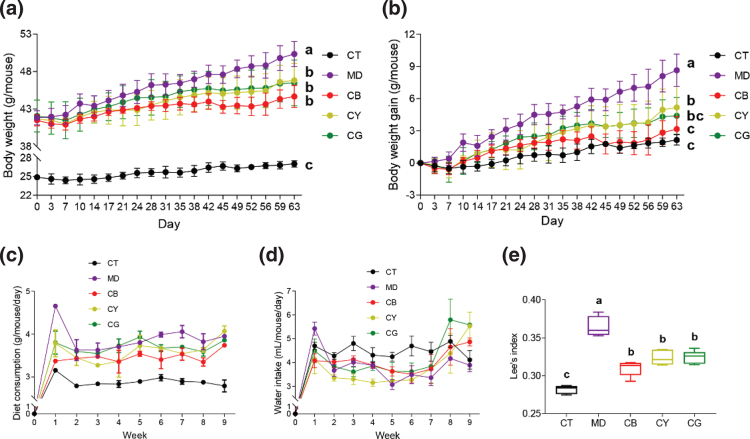
The effect of CMT on the changes of physical signs in mice. Mice were divided into C57BL/6J control (CT) group, ob/ob model (MD) group, *Citrus maxima* and black tea (CB), *Citrus maxima* and yellow tea (CY), and *Citrus maxima* and green tea (CG) treatment group. (a) Changes in body weight of mice during gavage treatment. (b) The weight gain value of the mouse body weight compared with the initial body weight during the gavage. (c) The amount of diet consumption during the gavage. (d) The amount of water intake during the gavage. (e) Lee’s index of mice at the end of gavage. The data are shown as the mean ± SD and *n* = 8. Different lowercase letters (a, b, and c) represent significant differences at the *P* < 0.05.

We gave the mice a continuous intragastric administration of CMT for 9 weeks and tested the physical signs of the mice. In the intragastric administration assay, compared with those of the C57BL/6J control (CT) mice, the body weight (Fig. 1a) and the weight gain (Fig. 1b) of the ob/ob model (MD) mouse increased significantly (*P* < 0.05). However, the weight and the weight gain of each CMT group were lower than that of the model group, and the reduction in the CB group was the most significant (*P* < 0.05). The average diet consumption and the average water intake of the mice per week are shown in Fig. 1c and d. Except for the control group, there was no significant difference in the average diet consumption of the mice between each tea group and the model group. There was no significant difference in the average water intake of mice between the groups. We used Lee’s index to evaluate the degree of obesity in mice. As shown in Fig. 1e, Lee’s index of the model group was significantly higher than that of the control group (*P* < 0.05). Compared with the model group, each tea group was significantly reduced (*P* < 0.05), but there was no significant difference between the tea groups. The above results indicated that CMT significantly inhibited the weight gain in mice, and CB had the best effect. CMT had no significant effect on the diet consumption and the water intake of mice.

### CMT regulates the storage of white fat

**Fig. 2:**
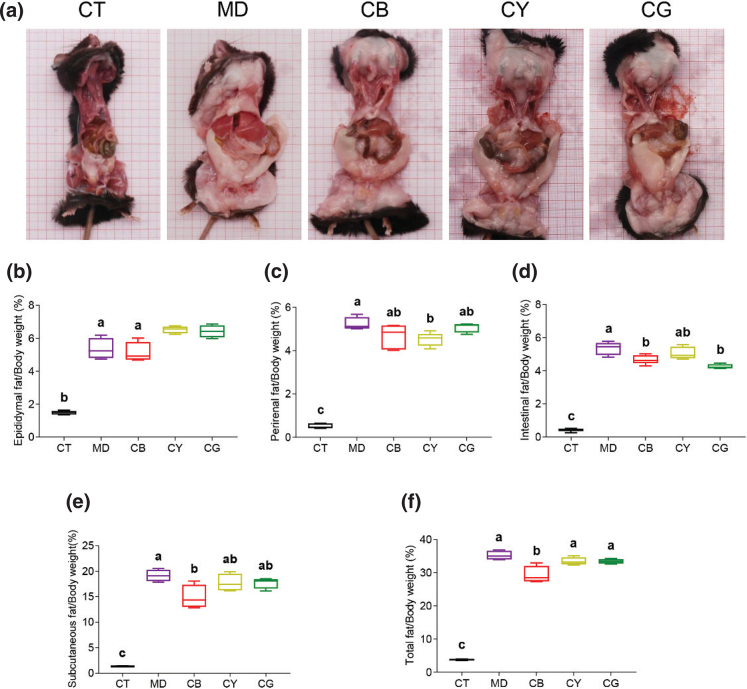
The effect of CMT on the storage of white fat in different parts of mice. (a) Overall view of mouse anatomy. (b) Epididymal fat index of the mice in each group. (c) Perirenal fat index in each group. (d) Intestinal fat index in each group. (e) Subcutaneous fat index in each group. (f) Total white fat index in each group. The fat index of different parts was expressed by the ratio of the weight of white fat to the weight of the mouse. The data in each group are shown as the mean ± SD and *n* = 8. Different lowercase letters (a and b) represent significant differences at the *P* < 0.05.

The overall appearance of the mouse anatomy is shown in Fig. 2a. Compared with the control group, the ob/ob model group had significantly more storage of white fat. Compared with the model group, the white fat of each tea group was reduced, and there was little difference between the tea groups. Then, we detected the accumulation of white fat in each part. CMT had no significant effect on the accumulation of epididymal fat (Fig. 2b). The perirenal fat percentage results showed that the CY group had a significantly lower perirenal fat percentage than the model group (Fig. 2c, *P* < 0.05). The intestinal fat percentage of the CB group or the CG group was significantly lower than that of the model group (Fig. 2d, *P* < 0.05). The subcutaneous fat percentage of the CB group was significantly lower than that of the model group (Fig. 2e, *P* < 0.05). Compared with that of the model group, the total fat percentage of the CB group was significantly reduced (Fig. 2f, *P* < 0.05), indicating that CB significantly inhibited the accumulation of total fat in mice. The accumulation of white fat and the white fat percentage of each part in the model group was significantly higher than those in the control group (*P* < 0.05). Our results suggested that the white fat accumulation and the corresponding index of the tea groups decreased compared with those of the model group.

### CMT reduces liver fat deposition and pathological inflammatory infiltration

**Fig. 3:**
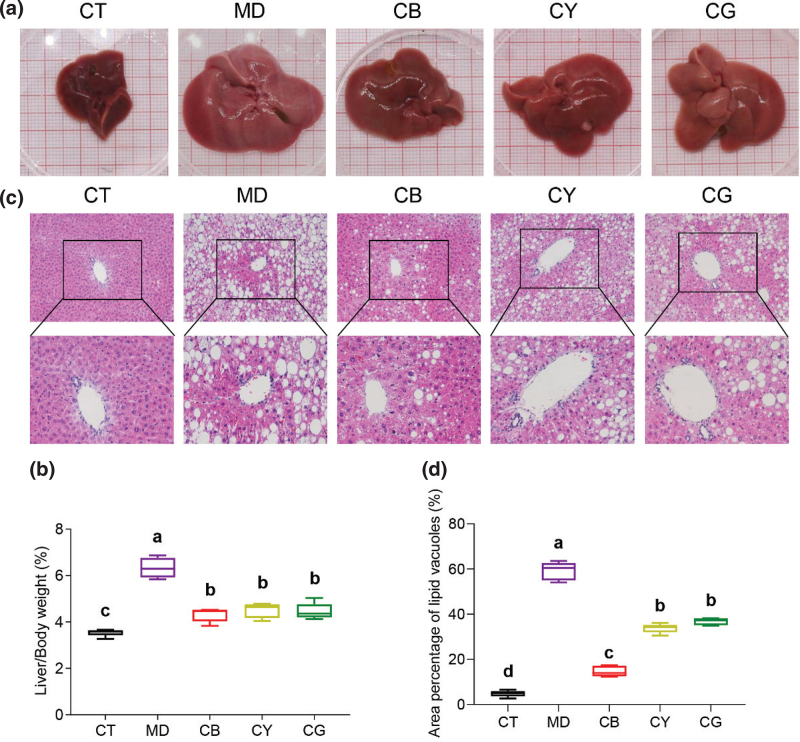
CMT relieves fatty liver and liver damage in mice. (a) The appearance of mouse liver. (b) Liver index expressed as the ratio of liver weight to mouse body weight. (c) The HE staining results of mouse liver sections (magnification: upper 200×, lower 400×). (d) The quantitative analysis results of lipid vacuoles in the HE staining image were analyzed by Image J, expressed as a percentage of the area of vacuoles in the field of view. The data in each group are shown as the mean ± SD and *n* = 8. Different lowercase letters (a, b, c, and d) represent significant differences at the *P* < 0.05.

To further study the effect of CMT on the physiological indicators of mice, we performed anatomical and pathological examinations on the liver of mice. As shown in Fig. 3a, the liver of the control group had a smooth surface, dark red color, and small volume. The liver surface of the model group was rough, had more white spots, and was whitish in color and larger in volume. Compared with that of the model group, the liver surface of each tea group was smoother, with fewer spots and smaller volume. The liver index results showed that the liver weight of the model group was significantly higher than that of the control group (*P* < 0.05), and each tea group was significantly lower than the model group (Fig. 3b, *P* < 0.05). The HE staining showed that compared with that of the control group, the liver of the model group showed a larger number of lipid vacuoles with a larger area, more severe cytoplasmic destruction, and more obvious inflammatory infiltration. Compared with the model group, the pathological characteristics of liver tissues in each tea group were significantly reduced (Fig. 3c). Among them, compared with the CY or CG group, the CB group had fewer lipid vacuoles, less cytoplasmic damage, and weaker inflammatory infiltration (Fig. 3c). The proportion of lipid vacuoles in the model group was significantly higher than that in the control group (*P* < 0.05), and that in each tea group was significantly less than that in the model group. The proportion of lipid vacuoles in the CB group was particularly significant (Fig. 3d, *P* < 0.05) in the three tea groups. The above results indicated that the ob/ob mice had an obvious fatty liver, and CMT with CB as the main type significantly reduced and alleviated excessive lipid deposition and inflammatory infiltration.

### CMT regulates serum and liver biochemical indicators

Furthermore, we tested the biochemical indicators including the blood lipids, FFA, ALT, and AST in mouse serums and liver tissues. The results was shown in Table 1. Compared with that in the control group, the content of TC, TG, LDL-C, and FFA in the model group increased significantly (*P* < 0.05), and the content of HDL-C was significantly reduced (*P* < 0.05). Compared with the model group, each tea group significantly reduced the content of biochemical indicators such as TC, TG, LDL-C, and FFA (*P* < 0.05) and significantly increased HDL-C (*P* < 0.05). Compared with the CY or CG group, the CB group had a significantly lower serum TC content (*P* < 0.05) and a significantly higher HDL-C content (*P* < 0.05). In addition, the serum and liver ALT and AST activity levels of the model group were significantly higher than those of the control group (*P* < 0.05). However, compared with those of the model group, the levels of these two biochemical indicators in each tea group were significantly reduced (*P* < 0.05). Therefore, we concluded that CMT (CB was the most significant) significantly improved the abnormal lipid indicators and liver damage in the ob/ob fatty liver mouse model.

**Table 1:** Changes of serum and liver physiological index levels in mice

Tissues & organs	Indicators	CT	MD	CB	CY	CG
Serum	TC (mmol/L)	12.545 ± 0.790^d^	35.003 ± 2.088^a^	21.964 ± 1.699^c^	26.836 ± 1.406^b^	29.034 ± 2.684^b^
	TG (mmol/L)	0.941 ± 0.093^c^	2.196 ± 0.230^a^	1.164 ± 0.088^bc^	1.365 ± 0.147^b^	1.446 ± 0.169^b^
	FFA (mmol/L)	0.972 ± 0.082^c^	2.222 ± 0.137^a^	1.299 ± 0.173^b^	1.338 ± 0.084^b^	1.327 ± 0.106^b^
	HDL-C (mmol/L)	3.244 ± 0.146^a^	2.192 ± 0.173^c^	3.235 ± 0.133^a^	2.832 ± 0.187^b^	2.855 ± 0.130^b^
	LDL-C (mmol/L)	0.475 ± 0.123^b^	1.772 ± 0.118^a^	0.691 ± 0.202^b^	0.616 ± 0.144^b^	0.532 ± 0.090^b^
	ALT (U/L)	6.536 ± 1.129^c^	92.872 ± 9.515^a^	25.876 ± 9.713^bc^	41.017 ± 14.707^b^	38.359 ± 14.808^b^
	AST (U/L)	13.093 ± 0.911^b^	39.638 ± 3.599^a^	15.354 ± 4.601^b^	17.108 ± 2.858^b^	19.673 ± 3.573^b^
Liver	TC (mmol/L)	0.253 ± 0.016^b^	0.473 ± 0.027^a^	0.268 ± 0.020^b^	0.292 ± 0.025^b^	0.312 ± 0.030^b^
	TG (mmol/L)	0.066 ± 0.011^b^	0.133 ± 0.013^a^	0.067 ± 0.005^b^	0.081 ± 0.010^b^	0.078 ± 0.010^b^
	FFA (mmol/L)	0.074 ± 0.005^c^	0.132 ± 0.007^a^	0.095 ± 0.006^b^	0.103 ± 0.010^b^	0.108 ± 0.009^b^
	HDL-C (mmol/L)	0.018 ± 0.001^a^	0.007 ± 0.001^c^	0.015 ± 0.001^b^	0.013 ± 0.001^b^	0.012 ± 0.001^b^
	LDL-C (mmol/L)	0.018 ± 0.004^b^	0.044 ± 0.002^a^	0.019 ± 0.002^b^	0.019 ± 0.001^b^	0.019 ± 0.002^b^
	ALT (U/L)	117.747 ± 8.314^d^	183.937 ± 9.376^a^	140.029 ± 4.105^c^	149.885 ± 5.328^bc^	158.581 ± 5.578^b^
	AST (U/L)	28.965 ± 2.426^b^	53.666 ± 1.714^a^	25.505 ± 5.104^b^	26.556 ± 5.389^b^	28.983 ± 3.099^b^

The data in each value are shown as the mean ± SD and *n* = 4. Different lowercase letters (a, b, c, and d) among the groups represent significant differences at the *P* < 0.05.

### CMT promotes phosphorylation of AMPK and ACC

**Fig. 4:**
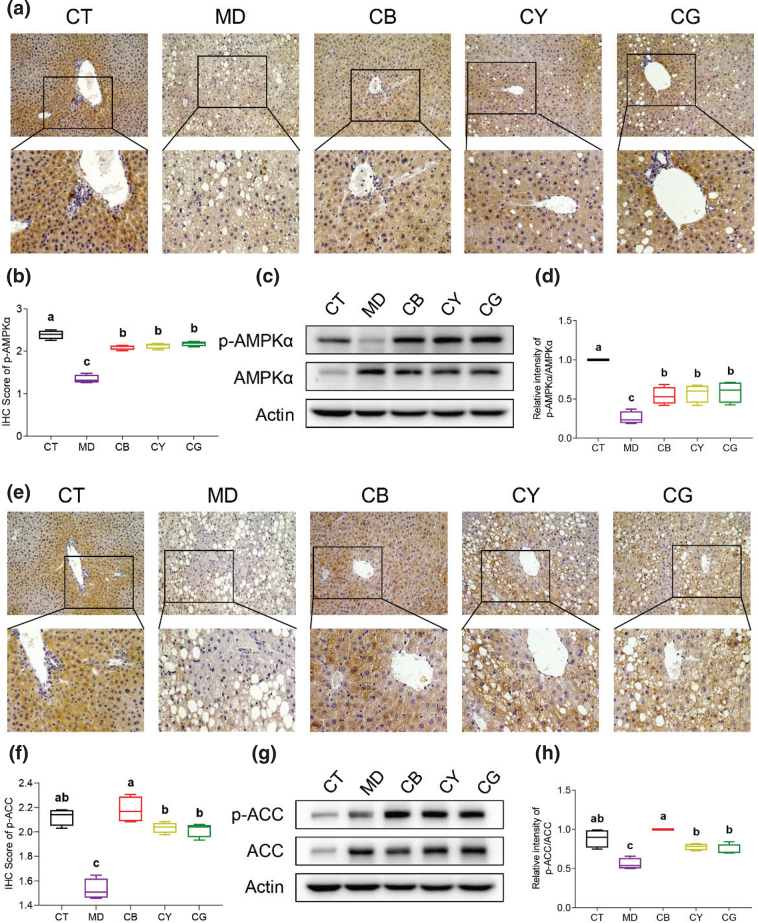
CMT regulates the protein phosphorylation level of AMPK and ACC. (a) The expression of p-AMPKα in liver tissue was detected by IHC (magnification: upper 200×, lower 400×). (b) Quantitative analysis results of 200× IHC pictures of p-AMPKα by Image J software. (c) Detection of the protein phosphorylation of AMPKα by western blot. (d) The ratio of p-AMPKα/AMPKα was measured by grayscale analysis by Image J software. (e) The expression of p-ACC in liver tissue was detected by IHC. (f) Quantitative analysis results of 200× IHC pictures of p-ACC by Image J software. (g) Detection of the protein phosphorylation of ACC by western blot. (h) Measure the ratio of p-ACC/ACC by grayscale analysis with Image J software. The data in each group are shown as the mean ± SD and *n* = 4. Different lowercase letters (a, b, and c) represent significant differences at the *P* < 0.05.

We analyzed the molecular mechanism of CMT in relieving non-alcoholic fatty liver. Previous studies have confirmed that AMPK was closely related to the regulation of lipid metabolism, and ACC was involved in fatty acid oxidation (20, 21). The immunohistochemistry (IHC) results of the mice liver showed that the expression of phosphorylated APMKα (p-AMPKα) was mainly distributed in the cytoplasm, and its expression level in the model group was significantly lower than that in the control group (*P* < 0.05). However, the expression level of p-AMPKα in each tea group was significantly higher than that in the model group (Fig. 4a and b, *P* < 0.05). The western blot results also showed that the p-AMPKα/AMPK level of each tea group was significantly upregulated (*P* < 0.05) compared with that of the model group (Fig. 4c and d). The IHC assays also confirmed that phosphorylated ACC (p-ACC) was distributed in the cytoplasm, and the p-ACC level in the mice model was rescued after the CMT treatment (Fig. 4e and f). The western blot analysis of p-ACC/ACC (Fig. 4g and h) was consistent with the conclusions of the IHC assays, and CB upregulated p-ACC most significantly in the three tea groups (*P* < 0.05). The above results indicated that CMT obviously activated the phosphorylation level of AMPK. Among the three types of CMT, CB activated the phosphorylation of ACC more significantly than the other two types (*P* < 0.05).

### CMT regulates FAS, CPT-1, and CD36 protein expression

**Fig. 5:**
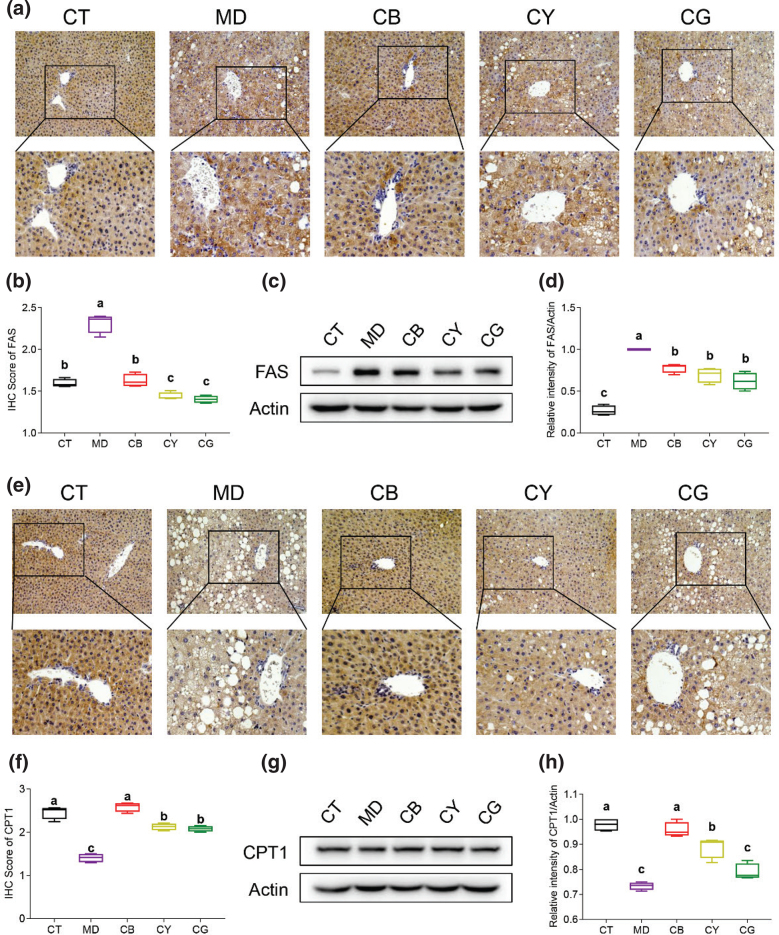
CMT regulates the expression levels of FAS and CPT-1. (a) The expression of FAS in liver tissue was detected by IHC (magnification: upper 200×, lower 400×). (b) Quantitative analysis results of 200× IHC pictures of FAS by Image J software. (c) Detection of FAS protein level by western blot. (d) Quantitative analysis of FAS level was performed by grayscale analysis by Image J software. (e) The expression of CPT-1 in liver tissue was detected by IHC. (f) Quantitative analysis results of 200× IHC pictures of CPT-1 by Image J software. (g) Detection of CPT-1 protein level by western blot. (h) Quantitative analysis of CPT-1 level was performed by grayscale analysis with Image J software. The data in each group is shown as the mean ± SD and *n* = 4. Different lowercase letters (a, b, and c) represent significant differences at the *P* <0.05.

FAS and CPT1 are the downstream proteins of AMPK/ACC signaling pathways, which regulate FAS and oxidation (22). The results of IHC (Fig. 5a and b) and western blot analysis (Fig. 5c and d) showed that FAS was mainly expressed in the cytoplasm, and the expression level in the model group was significantly higher than that in the control group (*P* < 0.05). However, the expression level of FAS in each tea group was significantly lower than that of the model group (*P* < 0.05), and that in the CY group and the CG group was more pronounced than that in the CB group. For CPT1, the IHC (Fig. 5e and f) and western blot analysis (Fig. 5g and h) results confirmed that the expression level of CPT1 in the model group was significantly downregulated compared with that in the control group (*P* < 0.05). The expression level of CPT1 in each tea group was significantly upregulated compared with that in the model group (*P* < 0.05), particularly in the CB group. These results suggested that CMT significantly inhibited the expression of FAS and activated the expression of CPT1 in the fatty liver.

**Fig. 6:**
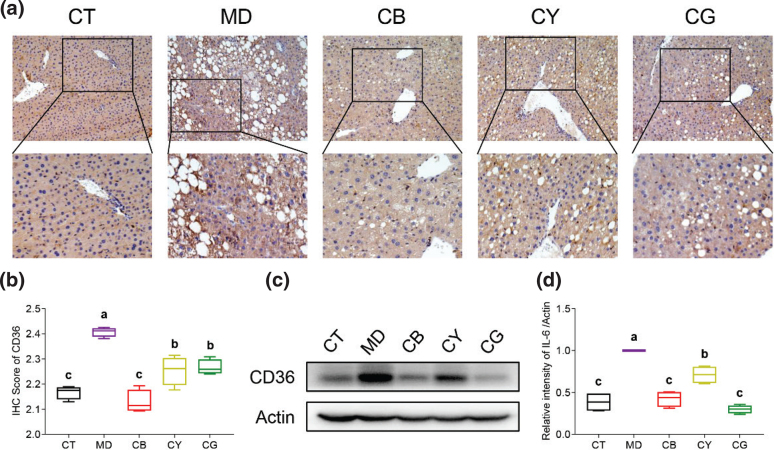
CMT regulates the protein expression levels of CD36. (a) The expression of CD36 protein in liver tissue was tested by IHC (magnification: upper 200×, lower 400×). (b) Quantitative results of 200× IHC pictures of CD36 by Image J software. (c) Evaluation of CD36 protein level by western blot. (d) Quantitative analysis of CD36 level was executed by Image J software. The data in each group are shown as the mean ± SD and *n* = 4. Different lowercase letters (a, b, and c) represent significant differences at the *P* <0.05.

Moreover, we have found that the FFA level in serum and liver tissue can be significantly alleviated by CMT (Table 1). Meanwhile, the CD36 protein is closed related to fatty acids metastasis. So, the protein expression level of CD36 was analyzed in liver tissue (Fig. 6). Compared with the CT group, the IHC (Fig. 6a and b) and western blot (Fig. 6c and d) results of CD36 protein expression in the MD group were markedly upregulated. Meantime, when NAFLD model mouse were treated with CMT, the expression level of CD36 was significantly suppressed. Interestingly, the CB group showed a better regulation effect.

### CMT changes inflammation-related protein expression

**Fig. 7:**
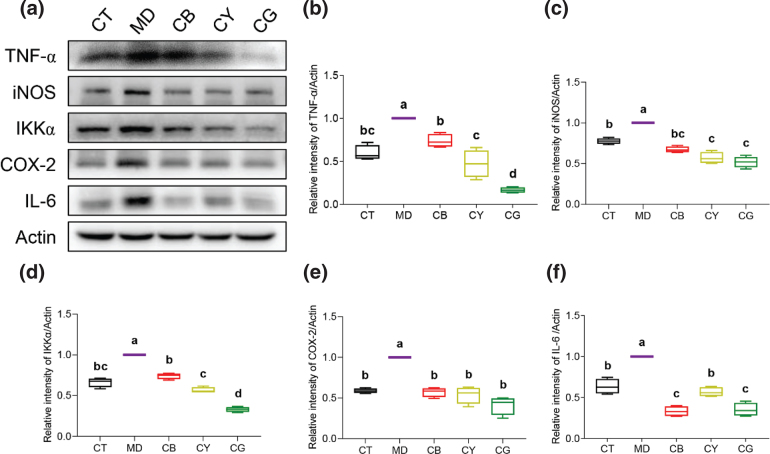
CMT regulates the expression of inflammatory factor to relieve hepatitis. (a) The protein expression levels of TNF-α, iNOS, IKKα, COX-2, and IL-6 in the liver tissues of each group were detected by western blot. (b–f) Quantitative analysis results of protein grayscale on TNF-α, iNOS, IKKα, COX-2, and IL-6 by Image J software. The data in each group are shown as the mean ± SD and *n* = 4. Different lowercase letters (a, b, c, and d) represent significant differences at the *P* < 0.05.

As the tea groups significantly improved the symptoms of hepatitis caused by the fatty liver, in order to reveal the corresponding molecular mechanism, we analyzed the protein expression of the related inflammatory factors in the liver tissue. The western blot (Fig. 7a) and the subsequent protein quantification results showed that the protein expression levels of TNF-α (Fig. 7b), iNOS (Fig. 7c), IKKα (Fig. 7d), COX-2 (Fig. 7e), and IL-6 (Fig. 7f) in the model group were significantly upregulated (*P* < 0.05), compared with those in the control group. Compared with the model group, each tea group showed a significant downregulation of the protein expression levels of the inflammatory factors (*P* < 0.05). Among them, CG and CY had a greater impact on TNF-α, iNOS, IKKα, and COX-2 than CB, while CB had a stronger regulation of the IL-6 protein expression level. Therefore, CMT significantly downregulated the protein expression level of the inflammatory factors, thereby reducing the inflammation caused by NAFLD.

## Discussion

The study revealed the alleviating effect of CMT on the symptoms of the NAFLD model mice. NAFLD is on the rise with the increasing number of obesity and diabetic populations and is trending toward globalization and younger individuals (23). Traditional Chinese diet therapy, including tea, has potential efficacy in improving liver function and relieving symptoms and has low side effects, showing broad prospects (24, 25). Studies have shown that the biologically active compounds in green tea had antioxidant, hypolipidemic, and anti-inflammatory effects, thereby reducing the risk of NAFLD (26, 27). Another tea, Kombucha KT, protected the liver cells from inflammation and fibrosis, which helped restore liver function in NAFLD mice (28). Our research showed a new drink of *Citrus maxima* cv. T. combined with green tea, yellow tea or black tea played an important role in restoring the liver function of NAFLD mice. The combination of CMT had a positive effect on improving fatty liver degeneration and damage.

The onset of NAFLD is mainly manifested as excessive fat accumulation in the liver (29). De novo fat synthesis is the process by which cells convert excess carbohydrates into fatty acids through acetyl-CoA. In the cytoplasm, acetyl-CoA is converted into malonyl-CoA by ACC and FAS (30). High levels of ACC and FAS in liver cells cause excessive fat synthesis, which is one of the causes of NAFLD (20). However, high levels of malonyl-CoA inhibit CPT1 and reduce fatty acid β-oxidation (31). The β-oxidation of fatty acids mediated by CPT1 is reduced in NAFLD (32). As a serine/threonine kinase, AMPK is a sensor of energy in cells, regulating the energy balance of cells and even the whole body. AMPK phosphorylates ACC and reduces ACC and malonyl-CoA levels (33). Therefore, AMPK inhibits fat de novo synthesis and increases mitochondrial fatty acid oxidation. The autophosphorylation of AMPK activates the upregulation of fat catabolism pathways (34). Metformin is considered to be one of the activators of AMPK, which reduces the accumulation of fat in the liver to treat NAFLD (35). Moreover, AMPK also directly downregulates the expression of lipidogenesis-related genes (including FAS and ACC) by phosphorylating the transcription regulator sterol regulatory element-binding protein 1c (SREBP-lc), which is a lipogenic transcription factor (36). Therefore, the activation of AMPK protects NAFLD by inhibiting the fat de novo synthesis and increasing the fatty acid oxidation. Our research showed that CMT activated the AMPK/ACC and AMPK/FAS signaling pathways in the liver to exercise its protective effect.

**Fig. 8:**
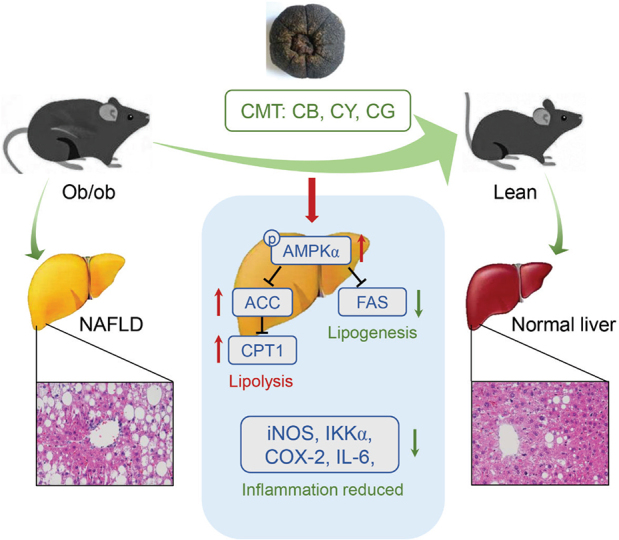
Graphical summary.

It is generally believed that tea is rich in polyphenols to promote the elimination of body fat and cholesterol and enhance the oxidation and decomposition of fatty acids (37). Dark tea extracts increase the serum HDL-C levels in mice and promote fat oxidation by inhibiting the activity of the obesity-related genes in the liver and the adipose tissue (38). The catechins in tea also reduce body fat accumulation by regulating the intestinal microbiota (39). The mechanism of tea in alleviating NAFLD has been previously studied, and it is closely related to AMPK signal activation. For example, Oolong tea extract increases the phosphorylation of AMPK and ACC and upregulates the expression of CPT1, thereby reducing the fat accumulation in mice. In high-fat-fed mice, a green tea extract improves the phosphorylation of AMPK and reduces the expression of ACC, FAS, and SREBP-1, thereby restoring normal liver metabolism and realizing the prevention of NAFLD (40). The polyphenols in green tea have a protective effect on the obese rat model of NAFLD and reduce liver fat production by upregulating the AMPK pathway (41). A theaflavin monomer significantly reduces the accumulation of lipid droplets in liver cells through the AMPK pathway (42). Our research suggested that CMT upregulated the phosphorylation of AMPK and ACC, thereby preventing abnormal lipid accumulation in the liver of ob/ob mice. Therefore, the use of tea extracts to target the AMPK/ACC and AMPK/FAS signaling pathways might become a new strategy for NAFLD treatment.

Moreover, studies have revealed the relationship between fat metabolism and fatty acids transfer, and the CD36 protein is a pretty important receptor protein for fatty acids entry liver cells (43). Under CMT treatment, our experiments not only confirmed that the FFA index in serum and liver tissue was markedly reduced in phenotype test but also certified that the CD36 expression level was significantly downregulated in mechanism analysis. More importantly, the regulation effect of CB in the three tea samples was better and stable.

NAFLD’s ‘two-hit’ theory is the consensus of the current disease mechanism. The first hit was liver steatosis, which may be mediated by the AMPK/ACC and AMPK/FAS pathways (44). The more important second hit is the inflammation caused by oxidative stress and the subsequent liver damage (45). The later stage of NAFLD is the activation of inflammasomes in hepatocytes, and then, the inflammatory factor storm appears, which is manifested by the excessive production of many inflammatory factors, such as TNF α, iNOS, IKKα, COX-2, and IL-6 (46, 47). Studies have shown that the activation of inflammasomes in the NAFLD liver leads to the expression of pro-inflammatory cytokines such as interleukin-1 beta (IL-1β) and interleukin-18 (IL-18) and promotes cell apoptosis by activating caspase-1 (48, 49). In NAFLD, a green tea extract rich in catechins restricts nuclear factor kappa-B (NF-κB) activation and reduces downstream inflammation (50). The polyphenols in Raw Bowl Tea reduce IL-1β, interleukin-4 (IL-4), IL-6, and other inflammatory factors to reduce the liver damage caused by NAFLD (51). Our data showed that CMT also inhibits the inflammatory factors TNF-α, iNOS, IKKα, COX-2, and IL-6 in ob/ob mice. Therefore, the inhibition of inflammation activation might be an effective treatment for NAFLD.

In short, CMT promotes fat oxidation and inhibits fat synthesis by activating the AMPK/ACC and AMPK/FAS signaling pathways. CMT also regulates the expression of the related inflammatory factors and reduces the inflammatory infiltration. In a practical sense, CMT effectively relieves NAFLD by reducing the lipid deposition and improving dyslipidemia.
